# The prevalence and associated factors of metabolic syndrome in Chinese aging population

**DOI:** 10.1038/s41598-020-77184-x

**Published:** 2020-11-18

**Authors:** Huisheng Ge, Zihui Yang, Xiaoyu Li, Dandan Liu, Yan Li, Yue Pan, Dan Luo, Xixi Wu

**Affiliations:** 1 Chengdu Women’s and Children’s Central Hospital, School of Medicine, University of Electronic Science and Technology of China, Chengdu, 611731 China; 2 Tsinghua Changgeng Hospital, Tsinghua University, Beijing, 102218 China; 3 Laboratory of Innovation, Basic Medical Experimental Teaching Center, Chongqing Medical University, Chongqing, 400016 China

**Keywords:** Endocrinology, Medical research

## Abstract

Metabolic syndrome (MetS) is hitting high notes in the aging society in China. However, the prevalence and associated factors in Chinese aging population lack clarity to some extent. In the present study, we projected to inquire into the prevalence of MetS and its associated factors by analyzing datasets downloaded from the China Health and Retirement Longitudinal Study (CHARLS). Data comprising age, gender, socioeconomic status, lifestyle and health behaviors as well as blood biomarkers were subjected to descriptive statistics followed by univariate logistic regression and multivariate logistic regression. The overall prevalence of MetS was 33.38% (95% CI 32.42–34.34%). With age augments, prevalence increased during 40–70 years, while declined in participants aged 70 years above. Females had 2.94 times of risks (95% CI 2.55–3.39, *P* < 0.001). Marital status and alcohol consumption contributed nothing to the suffering of MetS. Participants with GDP per capita > 10,000 RMB and a non-agricultural hukou sustained higher risks than other participants (*P* < 0.05). Participants under education of middle school suffered 1.16 times of risks than other level of education (95% CI 1.01–1.34, *P* < 0.05). Smokers, participants with high low-density lipoprotein (LDL) or hyperuricemia or high glycosylated hemoglobin HbA1c sustained increased risks (*P* < 0.05). In Chinese aging population, with the augment of age, the prevalence ascended in men, while descended in women and was interfered by socioeconomic status, lifestyle and health behaviors as well as blood biomarkers, but not marital status and alcohol consumption.

## Introduction

Metabolic syndrome (MetS), defined as a compound of visceral adiposity, hyperglycemia, dyslipidemia and hypertension by IDF (International Diabetes Federation) in 2005^1^, was hitting high notes due to its high prevalence and abominable long term outcome. Numerous studies had revealed the common role of MetS in children, adults and the aged, followed by a constellation of cardiovascular maladies^2,3^, prostate cancer^4^, breast cancer^5^ and massive other diseases. According to historical publications, MetS patients suffered 1.7 times of cardiac sudden death^6^, propensity to bone fracture^7^ and gout^8^, adding substantial burden to MetS patients and their households. A novel non-communicable disease, metabolic syndrome, had gradually been staged as a messy role globally. Hence, it is imperative to investigate the prevalence of MetS and its associated factors.

With the alterations in people's lifestyle and living standards improvement, incidence of MetS was treated by a rapid upstroke. The prevalence of MetS in different ethnic groups, different genders, different ages and different diagnostic criteria varied, more commonly seen in people over 50 years^9^. Previous study indicated that around 20–25% of adults worldwide suffered MetS^10^. In the Middle East, it was 37.4% based on IDF definition^11^ compared to 23.7% in America defined by Treatment of High Blood Cholesterol in Adults (ATP-III)^12^. Three historical studies in China revealed that this figure was 6.2%, 21.3% and 31.0% respectively^13–15^, which may be attributed to the diverse sampling population and diagnostic standards. In 2017, a project named China National Health and Nutrition Surveillance published their data of 11.0% in adults aged 18 years above^16^. From the studies aforementioned, we can see an inconsistent prevalence of each other. However, the trend of total prevalence significantly ascends with the augment of age^17^.

Currently, China houses 111 million (8.2% of the country population) elderly aged 65 years old above. This figure is estimated to reach 400 million citizens aged 65+ years old, 150 million of whom will be 80+ years old by 2050^18^. As the key population with high incidence of chronic diseases, the elderly population has high incidence rate, low awareness rate, low treatment rate, poor control effect, and more and more high coexistence rate of multiple diseases^19^. Prevalence of MetS in the aged is significantly higher than the overall prevalence, which surely will aggravate the living condition for them. However, no study with regard to the prevalence of MetS and its associated factors targeting the elderly is performed. This is exactly what we aim at in this project.

China Health and Retirement Longitudinal Study (CHARLS) investigated the baseline aging population including 28 provinces and 150 counties and collected a set of high-quality datasets. We download the datasets to scan the prevalence of MetS and associated factors including age, gender, marital status, gross domestic product (GDP) per capita, educational level, type of hukou, smoking, alcohol consumption, uric acid, low density lipoprotein (LDL) and glycosylated hemoglobin HbA1c. This dataset can provide researchers with chance to explore the association between metabolic syndrome and influence factor, which were stronger evidence-base medicine for clinical and public health interventions.

## Materials and methods

### Data

Data was downloaded from official website (https://charls.pku.edu.cn/) of the China Health and Retirement Longitudinal Study (CHARLS), an ongoing project operated by Peking University. Uncorking from 2008, this project updated its datasets in 2011, 2012, 2013, 2014, 2015. We selected the national baseline survey data in 2011–2012 to scan the prevalence and associated factors of MetS by downloading modules comprising Biomarker, Blood, Demographic background, Health status and functioning, Household income. After merging these modules by ID, further data cleansing was performed. A total of 8450 subjects were dropped from the original dataset because of containing missing value.

### Study design

**Figure 1:**
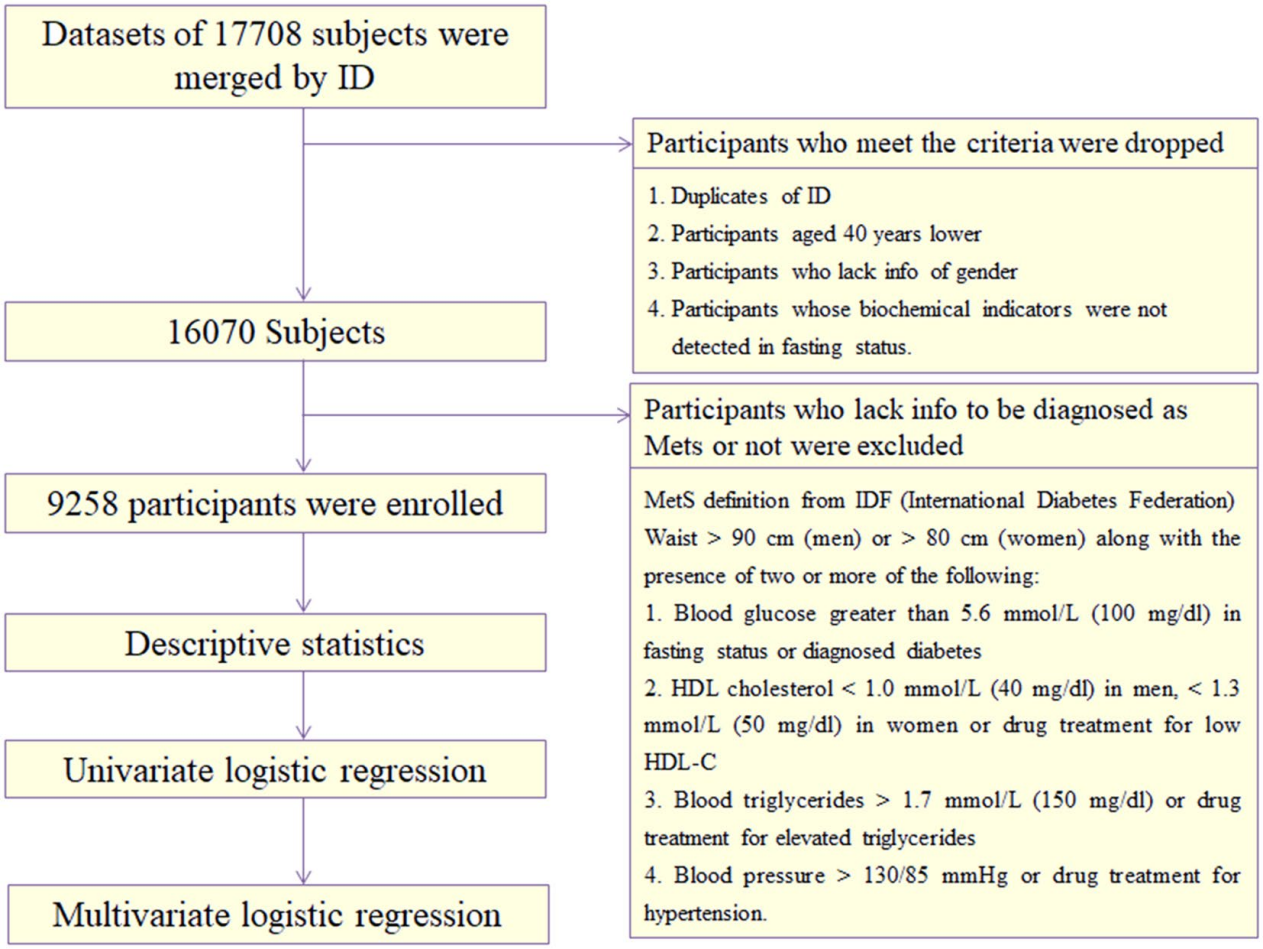
Flowchart of data cleansing.

Probabilities proportional to size (PPS) was adopted by CHARLS to collect datasets all over China. The project was stratified by regions, cities and gross domestic product (GDP). Further detailed account was shown in previous publications offered by CHARLS^20^. Finally, 150 counties in 28 provinces were selected to be investigated. In our study, a total of 17,708 subjects were first included. After clearing the duplicates of ID, participants aged 40 years lower and subjects who lacked info of gender and were not in fasting status, as well as subjects who lacked info to be diagnosed as MetS or not, 9258 participants in total were enrolled. Specific data cleansing processes were displayed in Fig. 1.

### Covariates

In our study, 11 associated factors were investigated including age, gender, marital status, GDP per capita, educational level, hukou, smoking, alcohol consumption, uric acid, low density lipoprotein (LDL), glycosylated hemoglobin (HbA1c). Age was stratified into 40–50 years, 50–60 years, 60–70 years and > 70 years. Gender was divided into men and women. Marital status was composed of married with spouse present and others, which included married but not living with spouse, separated, divorced, widowed and never married. GDP per capita consisted of < 1000 RMB, 1000–3000 RMB, 3000–5000 RMB, 5000–10,000 RMB and > 10,000 RMB. Educational level contained no formal education, elementary school, middle school, high school and college degree or above. Hukou meant the type of their ID card comprising agricultural hukou, non-agricultural hukou and others (unified hukou and no hukou). Smoking was divided into yes or no. Alcohol consumption comprised never drink, drink less than once a month and drink more than once a month. Uric acid was composed of hyperuricemia and non-hyperuricemia by a threshold concentration of 420 μmol/L for men and 357 μmol/L for women. LDL and HbA1c were all divided into normal and high by threshold values of 120 mg/dl and 6% respectively^21,22^.

### Definition of metabolic syndrome

Three principal definitions were adopted across the world defined by WHO, NCEP (National Cholesterol Education Program) and IDF (International Diabetes Federation)^23^. We selected the IDF definition to evaluate MetS due to its high specificity and sensitivity for Chinese and lower cutoff values, which benefited early identification and intervention. This definition consisted of five components including pivotally visceral adiposity, high blood glucose in fasting status and disordered lipid metabolism (elevator blood triglyceride plus katabatic high-density lipoprotein) as well as hypertension. Specific definition was shown in Fig. 1. In sensitivity analysis, NCEP ATP3 definition was used as the secondary one to validate the robust of result.

### Statistical analysis

The continuous data were described using mean ± SD, and the categories of data were proportions (%). Descriptive statistics were performed preliminarily to evaluate the data. Then, univariate logistic regression was executed to assess the outcome of MetS and associated factors. Then, significant factors were included into multivariate logistic regression model to explore the independent effects of its on MetS. Finally, adjusted odds ratio (OR) was calculated. All the analyses were performed using STATA 14.0 (Stata Corporation, College Station, TX, USA). *P* < 0.05 (two-sided) was considered as an indicator of statistical significance.

### Statements

The data was allowed to be downloaded and analyzed by the office of CHARLS. All methods were carried out in accordance with guidelines and regulations developed by the office of CHARLS and Chinese government. The CHARLS has been approved by institutional review boards at Peking University in June 2008. All participants were provided written informed consent for their participation in the survey.

## Results

### Sample characteristics of participants

**Figure 2:**
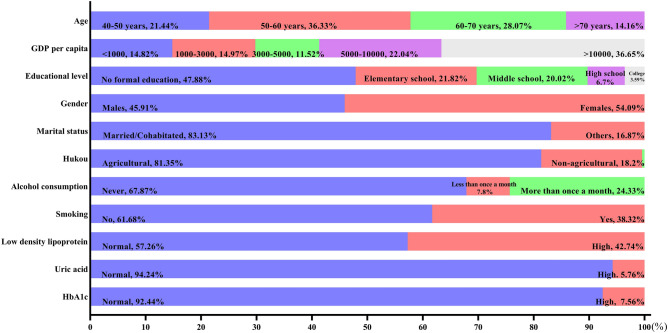
Sample characteristics of participants.

A total of 9258 participants aged 40 years above were enrolled with an average age of 59.50 years. Among them, 40–50 years, 50–60 years, 60–70 years and > 70 years accounted for 21.44%, 36.33%, 28.07% and 14.16% respectively (see Fig. 2). Percentage of women was higher than men (54.09% versus 45.91%). 83.13% subjects were married with spouse present, while the others were married but not living with spouse, separated, divorced, widowed and never married. For these participants, 14.82% suffered an income lower than 1000 RMB. 14.97%, 11.52%, 22.04% and 36.65% shared an income of 1000–3000 RMB, 3000–5000 RMB, 5000–10,000 RMB and > 10,000 RMB respectively. Nearly half of the participants (47.88%) received no formal education. Only 6.70% and 3.59% subjects were under education of high school and college degree or above. 81.35% participants were agricultural hukou, indicating they were more likely to settle in rural area compared to 18.20% non-agricultural hukou and other petty percentage subjects with no hukou or unified hukou. A total of 61.68% participants reported no history of smoking compared to 38.32% participants admitted. When referred to alcohol consumption, 67.87% reported no alcohol consumption, 7.80% admitted drinking but less than once a month and 24.33% drunk more than once a month. In our participants, 5.76% suffered hyperuricemia, 57.26% suffered higher low-density lipoprotein and 7.56% suffered higher HbA1c compared to 94.24%, 42.74% and 92.44% normal subjects respectively.

### Prevalence of MetS and its components

**Figure 3:**
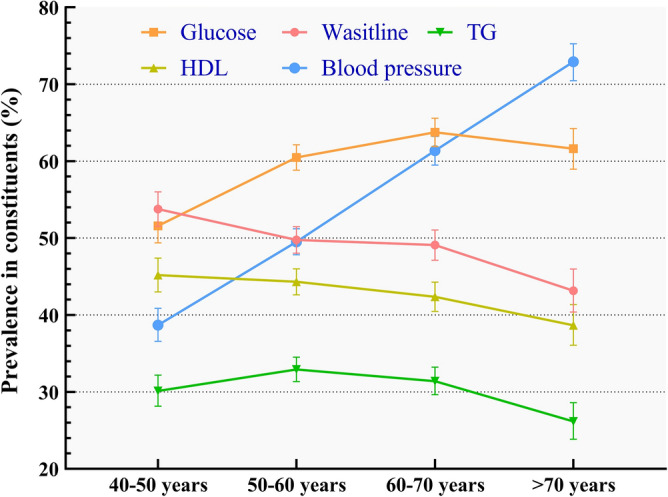
Prevalence of five constituents in different ages.

Definition of MetS comprised five components. Prevalence of the components under the IDF definition was displayed in Fig. 3. The overall prevalence of abdominal obesity, hyperglycemia, hypertriglyceridemia, low HDL cholesterol and hypertension was 49.54%, 59.66%, 30.94%, 43.15% and 53.83% respectively. It was significant that with the augment of age, incidence of hypertension ascended. However, prevalence of low HDL cholesterol and abdominal adiposity descended with senility. The trend in hyperglycemia and hypertriglyceridemia fluctuated.

The overall prevalence of MetS was 33.38%. This prevalence varied in different subgroups especially in gender, hukou, smoking, alcohol consumption, uric acid, LDL, and HbA1c. All the prevalence in subgroups was displayed in Table 1. Moreover, the total prevalence of MetS definition was 26.35% in NCEP-ATP3 definition.

**Table 1:** Prevalence of MetS in Chinese aging population.

Characteristics	Cases	Prevalence (%)	95% CI	Standard error (%)
Total	3090	33.38	32.42–34.34	0.49
**Age group**				
40–50	614	30.93	28.93–33.00	1.04
50–60	1140	33.90	32.32–35.52	0.82
60–70	930	35.78	33.96–37.65	0.94
> 70	406	30.97	28.52–33.53	1.28
**Gender**				
Men	862	20.28	19.10–21.52	0.62
Women	2228	44.49	43.17–45.87	0.70
**Marital status**				
Married with spouse present	2554	33.19	32.14–34.25	0.54
Others	536	34.31	32.00–36.71	1.20
**GDP per capita**				
< 1000	429	31.54	29.13–34.07	1.26
1000–3000	400	29.11	26.77–31.57	1.23
3000–5000	343	32.45	29.69–35.34	1.44
5000–10,000	655	32.38	30.37–34.45	1.04
> 10,000	1237	36.77	35.16–38.42	0.83
**Educational level**				
No formal education	1546	34.90	33.51–36.32	0.72
Elementary school	642	31.80	29.80–33.86	1.04
Middle school	593	32.02	29.93–34.18	1.08
High school	200	32.26	28.68–36.05	1.88
College degree or above	109	32.83	27.97–38.09	2.58
**Hukou**				
Agricultural	2384	31.66	30.62–32.72	0.54
Non-Agricultural	684	40.62	38.29–42.99	1.20
Others	22	52.38	36.91–67.41	7.80
**Smoking**				
No	2319	40.65	39.38–41.93	0.65
Yes	769	21.70	20.37–23.09	0.69
**Alcohol consumption**				
Never	2387	38.03	36.83–39.24	0.61
Less than once a month	195	27.05	23.92–30.42	1.67
More than once a month	506	22.49	20.81–24.26	0.88
**Uric acid**				
Non-hyperuricemia	2799	32.34	31.36–33.33	0.50
Hyperuricemia	249	47.07	42.83–51.35	2.17
**Low density lipoprotein**				
Normal	1469	37.49	35.99–39.02	0.77
High	1565	29.82	28.59–31.07	0.63
**HbA1c**				
Normal	2616	31.03	30.05–32.02	0.50
High	413	59.86	56.14–63.46	1.87

### Age and gender

**Figure 4:**
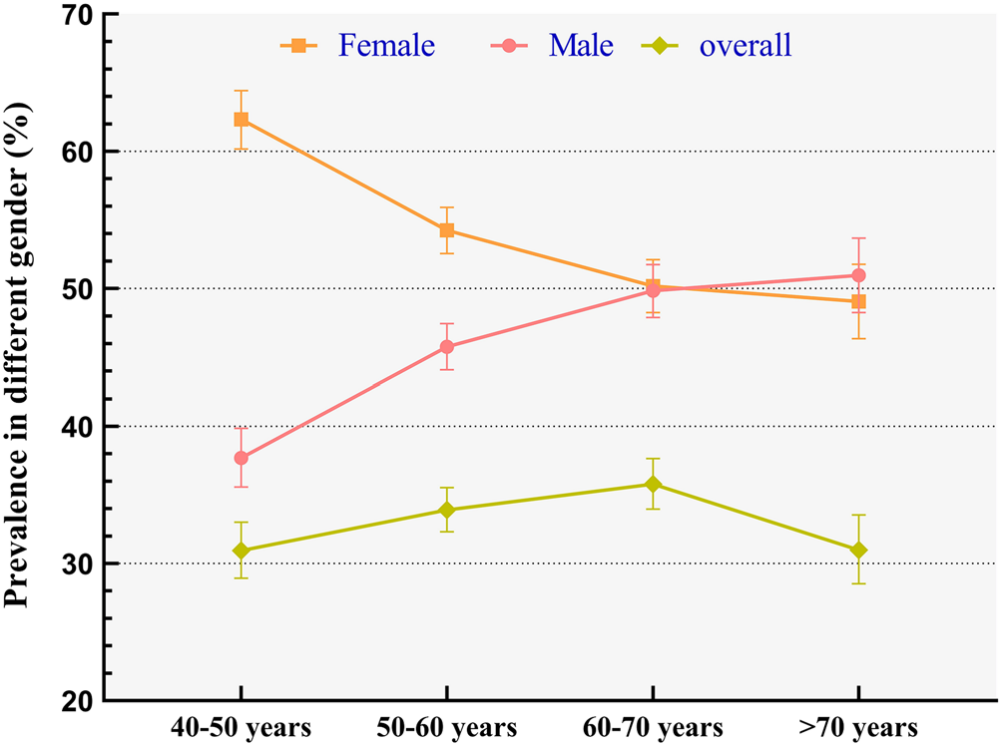
Prevalence of MetS in different age and gender.

**Figure 5:**
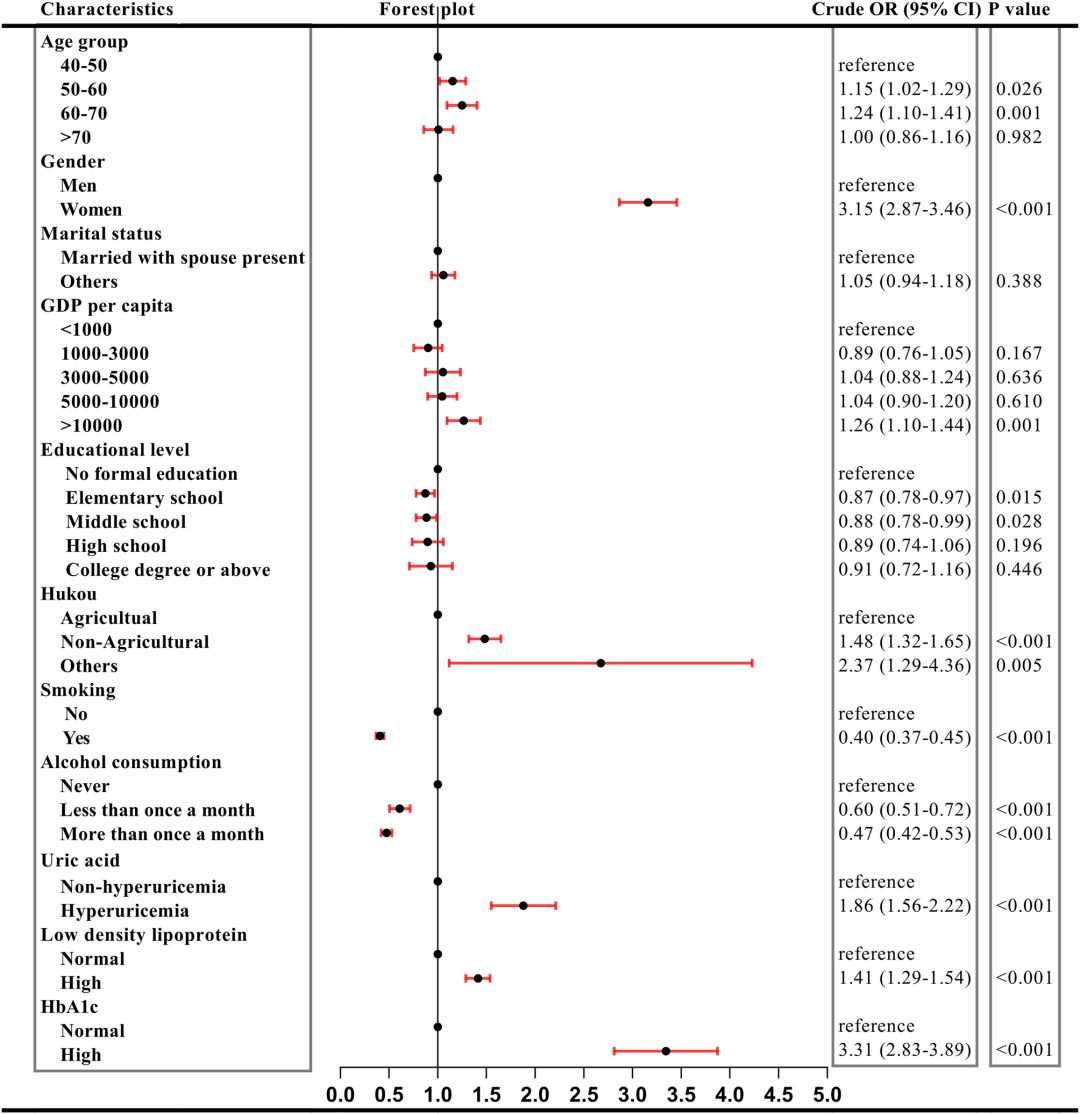
Results of univariate logistic regression.

**Figure 6:**
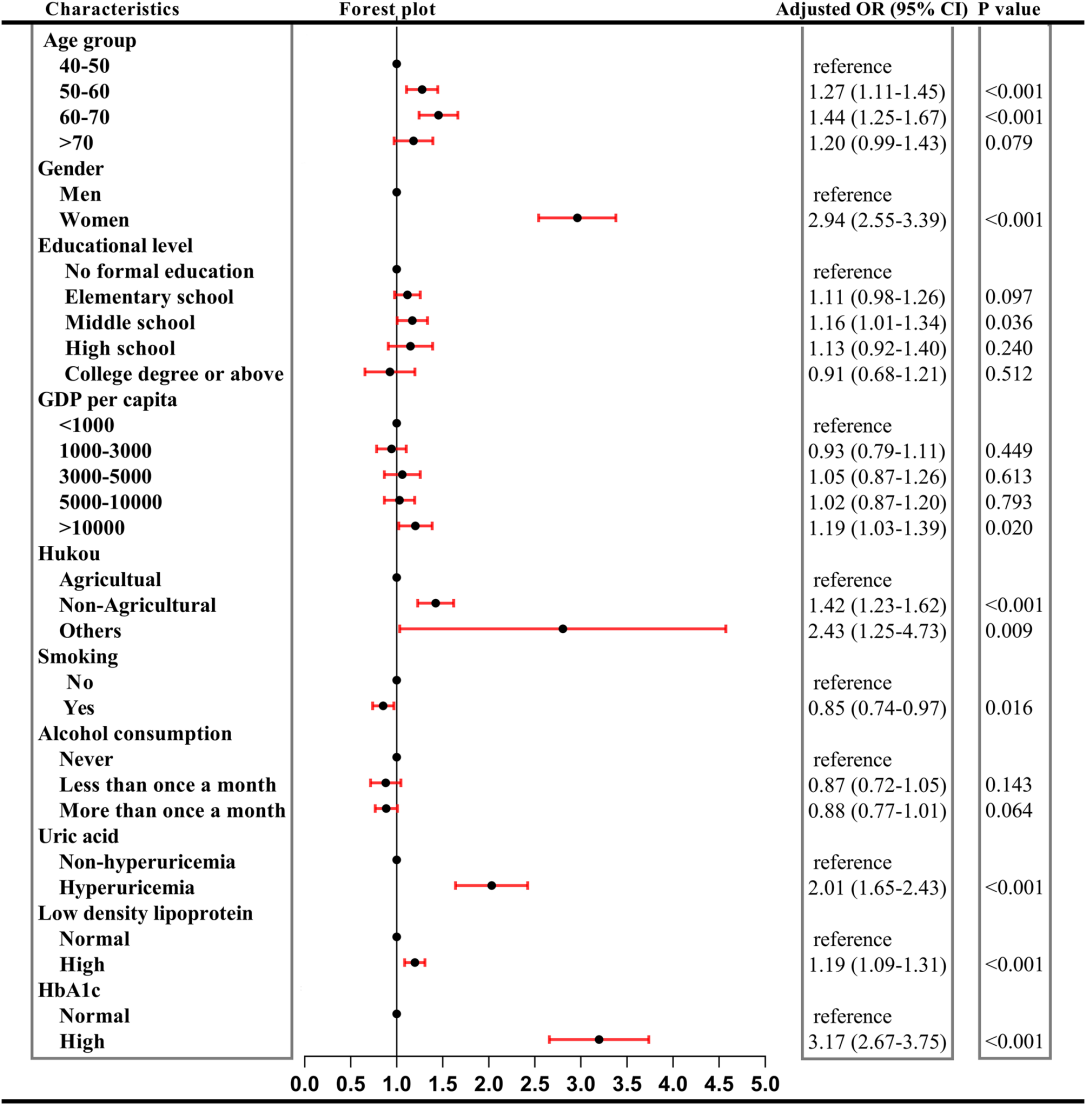
Results of multivariate logistic regression.

In our study, females sustained higher prevalence than male until 70 years above (see Fig. 4). Univariate logistic regression indicated 3.15 times of risks than men (Fig. 5). Overall risks in women were 2.94 times than men (95% CI 2.55–3.39, *P* < 0.001) after adjusted other covariant (see Fig. 6). It was obvious that with the augment of age, prevalence in men ascended, while in women descended. Regardless of gender, prevalence of MetS in 50–60 years and 60–70 years was higher than 40–50 years but this prevalence dropped in participants aged 70 years above (see Fig. 4). Univariate logistic regression reflected that with the augment of age, participants aged between 40–50 years and 50–60 years showed an increased propensity of MetS (*P* < 0.05), while participants aged > 70 years did not. After adjusted covariant, this tendency became more overt (see Fig. 6). It was noted that participants aged 70 years above still did not have a statistically significance, but the P value remained close to 0.05 (adjusted OR 1.20, 95% CI 0.99–1.43, *P* = 0.079).

### Marital and socioeconomic status

Discrepancy of prevalence was detected with regard to marital status in univariate logistic regression (crude OR 1.05, 95% CI 0.94–1.18, *P* > 0.05). Hence, no further multivariate logistic regression was performed. Socioeconomic status comprised GDP per capita, educational level and type of hukou. When the GDP per capita was higher than 10,000 RMB, the prevalence increased significantly. This elevation of incidence still was observed after multivariate logistic regression (adjusted OR 1.19, 95% CI 1.03–1.39, *P* < 0.05). Prevalence in participants with no formal education suffered highest prevalence of 34.90% (95% CI 33.51–36.32) compared to other subgroups. It was interesting to see that subjects with education of middle school sustained adjusted OR of 1.16 (95% CI 1.01–1.34, *P* < 0.05). Non-agricultural hukou sustained significantly higher prevalence than agricultural hukou (40.62%, 95% CI 38.29–42.99 vs. 31.66%, 95% CI 30.62–32.72). Subgroup named others could be ignored for its tiny sample size, only 42 participants. Adjusted OR indicated non-agricultural hukou sustained 1.41 times of risks than agricultural hukou (95% CI 1.23–1.62, *P* < 0.001).

### Lifestyle and health behaviors

Lifestyle and health behaviors included smoking and alcohol consumption. Participants who smoked had a high prevalence of MetS. Participants who smoked were prevalence of 21.70% (95% CI 20.37–23.09) versus prevalence of 40.65% (95% CI 39.38–41.93) for nonsmokers. Multivariate logistic regression revealed similar result that smokers enjoyed 0.85 times of risk than nonsmokers (95% CI 0.74–0.97, *P* < 0.05). This protective phenomenon did not recur in alcohol consumption. Participants who never drink possessed prevalence of 38.03% compared to 27.05% in drinkers less than once a month and 22.49% in drinkers more than once a month. Univariate logistic regression supported the same results where an increase in alcohol increase was associated with a lower risk of suffering from MetS (crude OR 0.60 drunk but less than once a month versus crude OR 0.47 in drunk but more than once a month). Nonetheless, the aforementioned results were cleared away when adjusted by covariates (adjusted OR 0.88, 95% CI 0.77–1.01, *P* > 0.05).

### Biochemical indicators

Biochemical indicators comprised uric acid, low density lipoprotein (LDL) and glycosylated hemoglobin HbA1c. In 2011, prevalence in non-hyperuricemia subgroup was 32.34% (95% CI 31.36–33.33), surging to 47.07% (95% CI 42.83–51.35) in hyperuricemia subgroup. When adjusted other variables, participants with hyperuricemia still sustained 2.01 times of risks than non-hyperuricemia (95% CI 1.65–2.43, *P* < 0.001) (Fig. 6). LDL and HbA1c connected with the definition of MetS including dyslipidemia and fasting plasma glucose augment. Both the two variables activated the risks of MetS as shown in Fig. 6. Participants with high LDL suffered 1.19 times of risks than normal LDL subjects (*P* < 0.001), while this risk increased to 3.17 times in high HbA1c participants compared to normal participants (*P* < 0.001).

## Discussion

Against the backdrop of aging society, enormous population countries especially for China are besetting by high prevalence of massive chronic diseases such as obesity^24^, diabetes^25^ and et al., which bring about heavy burden to the patients, households and society. Among these non-communicable chronic diseases, metabolic syndrome is a significant issue because it can be as a significant determinant of arterial aging, chronic kidney disease and multiple organ damage^26–29^. MetS was investigated in our study by downloading data from CHARLS, a data library with high quality, nationally representative datasets collecting from the aged across China.

It is calculated an overall MetS prevalence of 33.58% in our study under the definition of IDF. Previous publications display different results. Xi et al. reports a prevalence of 18.2% in participants aged 18 years above under the definition of IDF^13^. For the 40–59 years subgroup, this figure is 25.3% compared to 31.8% for the ≥ 60 years subgroup. Yu et al. revealed participants aged 35 years above suffered prevalence of 39.0% under the definition of NCEP-ATP III^14^. Under the definition of IDF, this figure dropped to 6.2% in participants aged 18–44 years. This may be attributed to age differences and the diverse definitions of MetS needing further unifying the diagnostic standards. Although the prevalence varies in different literatures, the trend with the age augments, prevalence increases remains clear, which is also seen in our study. But when participants are 70 years older, the tendency ascend. Due to the limitation of our cross-sectional design, we can’t assert this is because healthy people live longer or some other potential interpretations. The prevalence disparity in gender is universally observed with different results. Heiss et al. report women suffer higher prevalence^30^, while Al-Daghri et al. show a converse discovery^31^. This may partly be ascribed to regional and ethnic differences which needs further investigation.

Married with spouse present and other participants comprising married but not living with spouse, separated, divorced, widowed and never married share homogenous risks, which remain synonymous with preceding study indicating that widowed, divorced and single subjects suffer identical hazard with the married^32^. Besides marital status, socioeconomic status including educational level, GDP per capita and type of hukou is embroiled in the prevalence of MetS. Among the group, only middle school group sustains higher risks than others. Our study partly differs from Kim et al. In their cohort study, the risks of developing MetS in low education group (< 12 years) are higher than high education group (> 12 years)^33^. However, it is observed just in women, not in men. In China, middle school education equals to 9 years, which is lower than the 12 years cutoff value from Kim et al. Furthermore, participants with higher income suffer lower risks. However, this phenomenon only enters the spotlight when the income is over 10,000 RMB. The underlying reason may be similar with reasons for non-agricultural hukou suffers higher risks. High income often belongs to owners of non-agricultural hukou due to the income inequality in rural and urban areas leading to grave conditions for the suburb settler and low-income earners^34^. Further medical equality should be implemented.

Interestingly to see in the present study, smokers enjoy lower risks than nonsmokers, while no difference in drinkers and nondrinkers is spotted. Smoking acts as a protective role is seen in other diseases such as benign hyperplasia of prostate^35^. But for MetS, this result is contradictory to previous studies^36,37^. This finding may be explained for the fact that smoking and drinking were a close relationship, and the validation of smoking status was crude, which ignores specific number of tobacco use, and dose–response relationship should be examine in further study. As for alcohol consumption, risks are connected with how much participants drink^38^. But in our study, drinkers share same risks with nondrinkers.

In our study, urate concentration, LDL and HbA1C are positively corrected with MetS risks. Previous study indicates uric acid connects better with low HDL and high triglyceride. In low levels, uric acid correlates with parameters of metabolic syndrome linearly^39^. High concentration of LDL may increase hazard for prehypertension and hypertension, one constituent of MetS^40^. This may further lead to the augment of MetS. HbA1c reflects the glucose control in the past few months. It is overt that high HbA1c is 3.17 times of risks than normal HbA1c, which hints that glucose control plays a critical role in the increased hazard for the aged. Strict glycemic control should be implemented in the patients of hyperglycemia. A cohort study performed by Kawamoto et al. shows homogenous results^41^.

The present study has several strengths and defects. First, our study enrolls a large representative sample from Chinese aging population. The datasets collected by CHARLS are high-grade, which could reflect the authentic prevalence of MetS. Second, due to the limits of our cross-sectional design, we can’t evaluate the causality between the risk factors and MetS, needing further investigation. In our study, IDF definition, taking the regional and ethnic difference into account, is adopted to scan the prevalence of MetS. Another limitation was that important environmental factors including physical activity as well as dietary determinants such as energy intake did not evaluate and did not adjusted for covariates because of limited data.

## Conclusions

The overall prevalence of MetS was 33.38% in Chinese aging population in 2011. Among influence factors, the most effect indicators were HbA1c, gender and residence. The current study indicates that MetS still was severe public health issue among Chinese elder, and the key of primary prevention was aimed at high blood sugar, female and the population of urban. Further, a large longitudinal study should be conducted to examine these association.

## Data Availability

The data that support the findings of this study are available from China Health and Retirement Longitudinal Study (CHARLS) website (https://charls.pku.edu.cn/).

## References

[1] Alberti KG, Zimmet P, Shaw J (2005). The metabolic syndrome—A new worldwide definition. Lancet.

[2] Mottillo S (2010). The metabolic syndrome and cardiovascular risk a systematic review and meta-analysis. J. Am. Coll. Cardiol..

[3] Lakka HM (2002). The metabolic syndrome and total and cardiovascular disease mortality in middle-aged men. JAMA.

[4] Zhang JQ, Geng H, Ma M, Nan XY, Sheng BW (2015). Metabolic syndrome components are associated with increased prostate cancer risk. Med. Sci. Monit..

[5] Wani B, Aziz SA, Ganaie MA, Mir MH (2017). Metabolic Syndrome and Breast Cancer Risk. Indian J. Med. Paediatr. Oncol..

[6] Hess PL (2017). The metabolic syndrome and risk of sudden cardiac death: The atherosclerosis risk in communities study. J. Am. Heart Assoc..

[7] Yu CY, Chen FP, Chen LW, Kuo SF, Chien RN (2017). Association between metabolic syndrome and bone fracture risk: A community-based study using a fracture risk assessment tool. Medicine (Baltimore).

[8] Thottam GE, Krasnokutsky S, Pillinger MH (2017). Gout and metabolic syndrome: A tangled web. Curr. Rheumatol. Rep..

[9] Saklayen MG (2018). The global epidemic of the metabolic syndrome. Curr. Hypertens. Rep..

[10] Tanner RM, Brown TM, Muntner P (2012). Epidemiology of obesity, the metabolic syndrome, and chronic kidney disease. Curr. Hypertens. Rep..

[11] Delavari A, Forouzanfar MH, Alikhani S, Sharifian A, Kelishadi R (2009). First nationwide study of the prevalence of the metabolic syndrome and optimal cutoff points of waist circumference in the Middle East: The National Survey of risk factors for noncommunicable diseases of Iran. Diabetes Care.

[12] Ford ES, Giles WH, Dietz WH (2002). Prevalence of the metabolic syndrome among US adults: Findings from the third National Health and Nutrition Examination survey. JAMA..

[13] Xi B, He D, Hu Y, Zhou DP (2013). Prevalence of metabolic syndrome and its influencing factors among the Chinese adults: The China Health and Nutrition Survey in 2009. Prev. Med..

[14] Yu S, Guo X, Yang H, Zheng L, Sun Y (2014). An update on the prevalence of metabolic syndrome and its associated factors in Rural Northeast China. BMC Public Health.

[15] Sobko T (2014). Men in Macau SAR have higher prevalence in metabolic syndrome and among related metabolic components: A cross-sectional Macau Health Survey. BMC Public Health.

[16] He YN (2017). Prevalence of metabolic syndrome in Chinese adults in 2010–2012. Zhonghua Liu Xing Bing Xue Za Zhi.

[17] Firmann M (2008). The CoLaus study: A population-based study to investigate the epidemiology and genetic determinants of cardiovascular risk factors and metabolic syndrome. BMC Cardiovasc. Disord..

[18] Fang EF (2015). A research agenda for aging in China in the 21st century. Ageing Res. Rev..

[19] Li JH (2012). Awareness rate, treatment rate and control rate of dyslipidemia in Chinese adults, 2010. Zhonghua Yu Fang Yi Xue Za Zhi..

[20] Zhao Y, Hu Y, Smith JP, Strauss J, Yang G (2014). Cohort profile: The China Health and Retirement Longitudinal Study (CHARLS). Int. J. Epidemiol..

[21] Taguchi I, Iimuro S, Iwata H (2018). High-dose versus low-dose pitavastatin in Japanese patients with stable coronary artery disease (REAL-CAD): A randomized superiority trial. Circulation.

[22] Li Q, Li X, Wang J (2019). Diagnosis and treatment for hyperuricemia and gout: a systematic review of clinical practice guidelines and consensus statements. BMJ Open..

[23] Alberti KG, Eckel RH, Grundy SM (2009). Harmonizing the metabolic syndrome: A joint interim statement of the International Diabetes Federation Task Force on Epidemiology and Prevention; National Heart, Lung, and Blood Institute; American Heart Association; World Heart Federation; International Atherosclerosis Society; and International Association for the Study of Obesity. Circulation..

[24] Liu X (2018). Prevalence and influencing factors of overweight and obesity in a Chinese rural population: The Henan Rural Cohort Study. Sci. Rep..

[25] Yang L (2016). Prevalence of type 2 *Diabetes mellitus* among inland residents in China (2000–2014): A meta-analysis. J. Diabetes Investig..

[26] Scuteri A, Rovella V, Alunni Fegatelli D, Tesauro M, Gabriele M, Di Daniele N (2018). An operational definition of shats (systemic hemodynamic atherosclerotic syndrome): Role of arterial stiffness and blood pressure variability in elderly hypertensive subjects. Int. J. Cardiol..

[27] Nilsson PM, Laurent S, Cunha PG (2018). Characteristics of healthy vascular ageing in pooled population-based cohort studies: The global Metabolic syndrome and Artery Research Consortium. J. Hypertens..

[28] Scuteri A, Manolio TA, Marino EK, Arnold AM, Lakatta EG (2004). Prevalence of specific variant carotid geometric patterns and incidence of cardiovascular events in older persons. The Cardiovascular Health Study (CHS E-131). J. Am. Coll. Cardiol..

[29] Tesauro M, Canale MP, Rodia G (2011). Metabolic syndrome, chronic kidney, and cardiovascular diseases: Role of adipokines. Cardiol. Res. Pract..

[30] Heiss G (2014). Prevalence of metabolic syndrome among hispanics/latinos of diverse background: The Hispanic Community Health Study/Study of Latinos. Diabetes Care.

[31] Al-Daghri NM (2014). Gender-dependent associations between socioeconomic status and metabolic syndrome: A cross-sectional study in the adult Saudi population. BMC Cardiovasc Disord..

[32] Hosseinpour-Niazi S, Mirmiran P, Hosseinpanah F, Fallah-Ghohroudy A, Azizi F (2014). Association of marital status and marital transition with metabolic syndrome: Tehran lipid and glucose study. Int. J. Endocrinol. Metab..

[33] Kim I (2018). Educational disparities in risk for metabolic syndrome. Metab. Syndr. Relat. Disord..

[34] Gu H (2019). Measurement and decomposition of income-related inequality in self-rated health among the elderly in China. Int. J. Equity Health.

[35] Yoo S (2019). The impacts of metabolic syndrome and lifestyle on the prevalence of benign prostatic hyperplasia requiring treatment: Historical cohort study of 130454 men. BJU Int..

[36] Shin HS, Oh JE, Cho YJ (2018). The association between smoking cessation period and metabolic syndrome in Korean men. Asia Pac. J. Public Health.

[37] Park SH (2019). Smoking-related differential influence of alcohol consumption on the metabolic syndrome. Subst. Use Misuse..

[38] Sun K (2014). Alcohol consumption and risk of metabolic syndrome: A meta-analysis of prospective studies. Clin. Nutr..

[39] Cibickova L, Langova K, Vaverkova H, Kubickova V, Karasek D (2017). Correlation of uric acid levels and parameters of metabolic syndrome. Physiol. Res..

[40] Shimomura T, Wakabayashi I (2012). Associations of cardiovascular risk factors with prehypertension and hypertension in women. Blood Press.

[41] Kawamoto R, Akase T, Ninomiya D, Kumagi T, Kikuchi A (2019). Metabolic syndrome is a predictor of decreased renal function among community-dwelling middle-aged and elderly Japanese. Int. Urol. Nephrol..

